# Effect of portal access system and surgery type on surgery times during laparoscopic ovariectomy and salpingectomy in captive African lions and cheetahs

**DOI:** 10.1186/s13028-016-0199-2

**Published:** 2016-03-02

**Authors:** Marthinus Jacobus Hartman, Eric Monnet, Robert Murco Kirberger, Johan Petrus Schoeman

**Affiliations:** 1 Department of Companion Animal Clinical Studies, Faculty of Veterinary Science, University of Pretoria, Private Bag X04, Onderstepoort, Pretoria, South Africa; 2 Department of Clinical Sciences, Colorado State University, Fort Collins, CO USA

**Keywords:** Carbon dioxide, Laparoscopic, Ovariectomy, Salpingectomy, Single portal access system, SILS™, Sterilization, Surgery times

## Abstract

**Background:**

A prospective randomized study was used to compare surgery times for laparoscopic ovariectomy and salpingectomy in female African lion (*Panthera leo*) (n = 14) and cheetah (*Acinonyx jubatus*) (n = 20) and to compare the use of a multiple portal access system (MPAS) and single portal access system (SPAS) between groups. Two different portal techniques were used, namely MPAS (three separate ports) in lions and SPAS (SILS™ port) in cheetahs, using standard straight laparoscopic instruments. Portal access system and first ovary was not randomized. Five different surgery times were compared for the two different procedures as well as evaluating the use and application of MPAS and SPAS. Carbon dioxide volumes for lions were recorded.

**Results:**

In adult lionesses operative time (OPT) (*P* = 0.016) and total surgical time (TST) (*P* = 0.032) were significantly shorter for salpingectomy compared to ovariectomy. Similarly in cheetahs OPT (*P* = 0.001) and TST (*P* = 0.005) were also shorter for salpingectomy compared to ovariectomy. In contrast, in lion cubs no difference was found in surgery times for ovariectomy and salpingectomy. Total unilateral procedure time was shorter than the respective bilateral time for both procedures (*P* = 0.019 and *P* = 0.001) respectively and unilateral salpingectomy was also faster than unilateral ovariectomy (*P* = 0.035) in cheetahs. Port placement time, suturing time and TST were significantly shorter for SPAS compared to MPAS (*P* = 0.008). There was, however, no difference in OPT between SPAS and MPAS. Instrument cluttering with SPAS was found to be negligible. There was no difference in mean volume CO_2_ required to complete ovariectomy in lions but the correlation between bodyweight and total volume of CO_2_ in lions was significant (r_s_ = 0.867; *P* = 0.002).

**Conclusions:**

Laparoscopic salpingectomy was faster than ovariectomy in both adult lions and cheetahs. Using SPAS, both unilateral procedures were faster than bilateral procedures in cheetahs. Placement and suturing of SPAS in cheetahs was easier and faster compared to three separate ports in lions and lion cubs. The use of standard straight instruments during SPAS did not prolong surgery. Surgery was faster in cubs and CO_2_ required for laparoscopic sterilization in lions could be determined. Predictable surgery times and CO_2_ volumes will facilitate the accurate planning and execution of surgery in lions and cheetahs.

## Background

The natural habitat of wildlife species, including wild felids, is under constant threat of human invasion, causing a decline in wildlife numbers worldwide. In contrast, commercial farming of African lions in South Africa and captive keeping of cheetahs in Namibia are posing unique challenges to these countries. Over the past decade the exploitation of canned hunted African lions in South Africa resulted in stricter legislative control [1]. This, coupled with a worldwide economic recession resulted in excessive numbers of lions on commercial farms, which necessitated radical population control at the time. Increasing numbers of free ranging lions in smaller parks in South Africa also pose a growing threat to antelope populations, since natural predation of these lions quite often do not exist. In Namibia, the number of cheetahs held as captive bred or rescued individuals, grew to proportions where Namibian authorities currently insist on permanent sterilization of all female large carnivores, including cheetahs, in captivity [2]. Consequently, surgical sterilization may provide a potential solution to both of these situations. Wildlife surgery poses considerable and unique challenges to the veterinary team. Factors such as procurement of sterile facilities, equipment and the availability of drugs and consumables need to be considered when planning surgical sterilization of especially wild felids outside the normal theatre environment. Moreover, surgery time becomes an important factor for sterilization projects of these wild felids kept on farms or in conservation centers and especially under free ranging conditions.

Minimally invasive surgical techniques present specific advantages over conventional surgical approaches. Lower post-operative infection rates [3], early return to normal activity [4] and lower post-operative pain [5–7] are some examples, especially in wild felids. Additionally, surgical site complication rates are also expected to be lower with minimally invasive surgery. Shorter surgery times, in particular, are becoming an increasingly important consideration in all forms of surgery. As a result, various laparoscopic studies lead to burgeoning veterinary literature on this aspect, especially the reduction of number of ports [8–13]. However, although a reduction in the number of ports used for laparoscopy influences tri-angulation [14] and surgical comfort, it also holds various advantages [9, 11]. Therefore, portal configurations for laparoscopic surgery have evolved considerably over recent years [8, 9, 11, 13, 15–19]. Three separate ports have been used during the sterilization of lions [17] whereas single incision laparoscopic surgery (SILS) has been used in cheetahs [18], two tigers [12] and two leopards [20]. Concerning requirements for laparoscopic wildlife surgery, consumables such as carbon dioxide (CO_2_) volumes have been determined in the cheetah [18] and leopard [20] however, these volumes for laparoscopic surgery in other species are unknown.

The purpose of this study was to report and compare various surgery times associated with laparoscopic ovariectomy and salpingectomy and to compare a multiple portal access system (MPAS) and a single portal access system (SPAS) during sterilization of African lions and cheetahs. In this study, MPAS refers to the use of three separate ports and SPAS to the use of a SILS port. It was hypothesized that laparoscopic salpingectomy would be faster than ovariectomy in lions and cheetahs respectively, that sterilization would be faster in cheetahs compared to lions for each respective procedure and that instrument cluttering will complicate surgery and prolong surgery time for SPAS. Moreover, required volume of CO_2_ in lions needs to be determined.

## Methods

### Animals

Three groups of animals were included, adult lionesses (n = 9), lion cubs (n = 5) and adult cheetahs (n = 20). Lions under the age of 24 months are classified as small cubs however, for purposes of this study they were merely referred to as cubs. The lions were from a lion farm in South Africa and cheetahs from two different conservation centers in Namibia. On the lion farm, an old school building was converted into a theatre complex during the two days preceding the research project and consisted of a separate surgical preparing area, ultrasound room and operating room. Both cheetah centers had well equipped theatre complexes on site. A need for permanent sterilization of African lions on lion farms in South Africa arose and the captive cheetahs in Namibia had to comply with amended legislation. Patients were allocated to two procedures namely ovariectomy and salpingectomy by using a randomization table. Two different portal techniques were used, namely MPAS (three separate ports) in all lions and SPAS (SILS™ Port, Covidien™) in all cheetahs, using standard straight laparoscopic instruments only, however the portal access system was standard for each group of patients. The right ovary was routinely operated first and the left ovary second. Determined patient data were body weight and age. The project was approved by the Animal Ethics Committee and Research Committee of the University of Pretoria (protocols numbers v051-10 and v014-14).

### Anaesthesia

The anaesthetic protocol, surgical techniques and equipment were those previously described for lions [17] and cheetahs [18]. Briefly, lionesses were immobilized using a combination of tiletamine and zolazepam (Zoletil^®^, 100 mg/ml, Virbac, Halfway House, South Africa) or tiletamine and zolazepam and medetomidine (Domitor^®^, 1 mg/ml Pfizer Animal Health, Sandton, South Africa) and cheetahs, tiletamine and zolazepam (Zoletil^®^, 100 mg/ml, Virbac, Halfway House, South Africa) and medetomidine at doses of 1.2 and 0.03 mg/kg respectively, administered intramuscularly either by a remote projection system or injected by hand in cheetahs that were trained to enter a squeeze cage. Once the animal was suitably immobilized, it was transported to the surgery facility. Intravenous propofol (Propofol^®^, 10 mg/ml, Fresenius-Kabi, Halfway House South Africa) at 4–6 mg/kg was given to effect to facilitate intubation in lionesses. Intravenous fluid 10 ml/kg/h Ringer’ Lactate (Intramed Ringer-Lactate Solution^®^, Fresenius-Kabi, Port Elizabeth, South Africa) was administered until extubation. Patients were connected to a semi-closed, rebreathing anaesthetic circuit during preparation and maintained with a mechanical ventilator on a closed circuit on isoflurane gas (Isofor^®^, Safeline Pharmaceuticals, Weltevredenpark, Roodepoort, South Africa). Morphine sulphate at 0.3 mg/kg (Morphine, 10 mg/ml, Fresenius Kabi, Port Elizabeth, South Africa) was administered subcutaneously after intubation for intraoperative analgesia and 1.5 ml of ropivacaine (Naropin^®^, 7.5 mg/ml, AstraZenaca Pharmaceuticals, Sunninghill Johannesburg, South Africa) subcutaneously at the surgical incision site. Meloxicam 0.3 mg/kg (Metacam^®^, 5 mg/kg, Boehringer Ingelheim, Pine Avenue, Randburg, South Africa) was administered subcutaneously prior to anaesthetic recovery for post-operative analgesia. All surgeries were performed simultaneously by two surgeons (EM and MH). After completion of surgery, patients were weaned off the ventilator, and re-placed in transporting crates where they were extubated. Cheetahs were allowed to recover inside the crates and lions in their over-night houses under constant supervision of the anaesthetic team and care takers. During the first 24 h after surgery, patients were monitored intensively for any abnormalities associated with habitus, appetite, wound dehiscence, and haemorrhage also frequently during the following months.

Surgery times were recorded during both these studies. Abdominal wall thickness was measured ultrasonographically from the skin surface to the peritoneal line immediately cranial to the umbilicus. The sizes of ovaries removed were measured in length, width and height using a caliper and ovarian volume was then determined using a prolate ellipsoid formula [21]. Periovarian structures were evaluated ultrasonographically and recorded. Ovarian pedicle fat content and uterine tube and mesosalpinx morphology were subjectively compared laparoscopically. In lions, the first cannula port was placed using the modified Hasson technique [17] and subsequent ports routinely. For SPAS, the SILS port was removed for retrieval of all ovaries however, for MPAS small ovaries were retrieved through the 12 mm port and larger ovaries removed together with the cannula after enlargement of the incision.

### Surgery

**Fig. 1:**
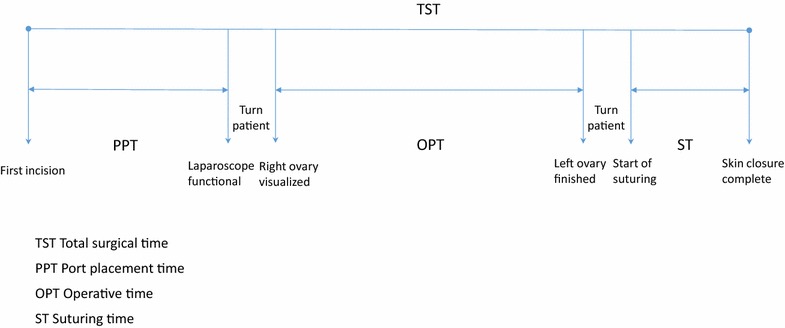
Surgery times for laparoscopic ovariectomy and salpingectomy

Four different surgery times were recorded in minutes (Table 1). Port placement time (PPT) was determined from first incision to time when the laparoscope was functional, operative time (OPT) from the first time the right ovary was laparoscopically visualized to the time surgery on the left ovary was finished, suturing time (ST) from start to end of suturing and total surgical time (TST) was calculated from the first incision to completion of skin closure. For the MPAS, end of PPT was defined at completion of the 3rd cannula placement and for SILS time when the laparoscope was functional. Therefore, PPT included time to insufflate the peritoneal cavity to 13 mmHg, as well as the introduction of a laparoscope, for both techniques. Time to turn patients from dorsal recumbency to left oblique and time from last (left) ovary to start of suturing (which included turning the patient from right oblique back to dorsal recumbency) were not included in PPT, OPT or ST but were included in TST. From the data set, the total surgical time for unilateral procedure (TUPT) for ovariectomy and salpingectomy was extrapolated (Table 2). For unilateral ovariectomy, the surgery time between removal of the first and second ovary was subtracted and for salpingectomy time between completion of the first and second salpingectomy (Fig. 1).

Five repetitive cases in similar-sized animals for each of the two different portal techniques (MPAS and SPAS) were selected and compared for time and ease of application during the two different surgical procedures (three ovariectomies and two salpingectomies for each technique). This was after completion of a learning curve of at least four cases in both procedures in each respective species. The total volume of CO_2_ required, to achieve and maintain an intra-abdominal pressure of 13 mmHg throughout surgery, was recorded in all lions.

Statistical analysis was performed using SPSS v.17 (IBM, New York, USA) statistical software package. Normally distributed data were summarized as mean and standard deviation and non-parametric data as median and interquartile range (IQR) according to Tukey’s Hinges. For body weight and age in cheetahs the Kolmogorov–Smirnov test was used to test for normality and Shapiro–Wilk for volume of CO_2_ in lions. The independent samples Student *t* test was used to compare means and Levene’s test to test for equal variances. For body weight and age in lions and all surgery times the Mann–Whitney U Test was used to compare medians. Pearson’s correlation was used to determine the correlation between body weight and TST in cheetahs and Spearmann’s rank order for correlation between body weight and TST and volume of CO_2_ in lions. A simple linear regression model was used to determine the relationship between bodyweight and volume of CO_2_ and TST in lions. The significance level was set at *P* < 0.05.

## Results

All patients returned to normal appetite and behavior within 18 h of surgery. In lions, no post-surgical complications were encountered. One cheetah engaged in mild self-mutilation of the tail and a leg in the recovery period that ceased once the animal fully-recovered. One cheetah developed visible signs of mild inflammation around the surgical incision that resolved within 24 h. None of the patients developed incisional hernias. No surgically related complications were reported three months after surgery.

In adult lions, there was no significant difference in median body weight or age between the ovariectomy (145 kg [130–160] and 9.0 years [6–9]) and salpingectomy (140 kg [134–141] and 9.0 years [9, 10]) groups. All five lion cubs were 8-months-old and there was no significant difference in median body weight for the ovariectomy (40.0 kg [39.5–41]) and salpingectomy (43.5 kg [42–45]) cubs. For cheetahs there was also no significant difference in mean body weight or age between the ovariectomy (32.6 ± 2.0 kg and 11.3 ± 2.9 years) and salpingectomy (33.3 ± 3.6 kg and 11.1 ± 2.7 years) groups.

Surgery times are summarized in Tables 1 and 2. In adult lionesses there was no significant difference in PPT or ST between ovariectomy and salpingectomy however, OPT was significantly shorter for salpingectomy compared to ovariectomy (*P* = 0.016) and so was TST (*P* = 0.032). In lion cubs salpingectomy was faster than ovariectomy but not significantly so. In cheetahs, there was no significant difference in PPT or ST however, OPT was also significantly shorter for salpingectomy compared to ovariectomy (*P* = 0.001) and so was TST (*P* = 0.005). TST for both procedures were significantly shorter in cubs compared to adult lions (*P* = 0.001) and both procedures were significantly faster in cheetahs compared to lion cubs (*P* = 0.001). The correlation between bodyweight and TST in lions (r_s_ = 0.777; *P* = 0.001; r^2^ = 0.599) was significant but not in cheetahs.

**Table 1:** Surgery times for bilateral ovariectomy and salpingectomy in lions and cheetahs in minutes

Group	n	Procedure	PPT		OPT		ST		TST	
			Med	IQR	Med	IQR	Med	IQR	Med	IQR
Adult Lions	4	Ove	16	13–21	31	25–37	13.5	13–16	67	55–78
	5	Salp	18	15–18	9	9–10	11	10–14	47	41–47
Lion cubs	3	Ove	11	10–11	9	9–10	13	12–14	36	35–37
	2	Salp	7	6–8	6	5–7	11.5	11–12	29	28–30
Cheetahs	10	Ove	4	3–5	9.5	8–11	9	8–9	24	23–26
	10	Salp	4	3–4	5	4–6	8.5	8–9	19.5	17–20

*n* number of procedures, *PPT* port placement time, *OPT* operative time, *ST* suturing time, *TST* total surgical time, *Med* Median, *IQR* Inter quartile range, *Ove* ovariectomy, *Salp* salpingectomy

**Table 2:** Total surgery time for unilateral ovariectomy and salpingectomy in lions and cheetahs in minutes

Group	n	Procedure	TUPT	
			Med	IQR
Adult lions	4	Ove	46	42–57
	5	Salp	39	35–43
Lion cubs	3	Ove	30	30–31
	2	Salp	25.5	24–27
Cheetahs	10	Ove	19	17–20
	10	Salp	16	15–18

*n* number of procedures, *TUPT* total unilateral procedure time, *Med* Median, *IQR* Inter quartile range, *Ove* ovariectomy, *Salp* salpingectomy

Comparing the two unilateral procedures, TUPT for ovariectomy and salpingectomy was not statistically different in adult lions or lion cubs, however, unilateral salpingectomy was significantly faster compared to ovariectomy in cheetahs (*P* = 0.035). Comparing TUPT of either ovariectomy or salpingectomy to TST for the respective bilateral procedure within each animal group, unilateral salpingectomy in cheetahs was significantly faster than bilateral salpingectomy (*P* = 0.019), and unilateral ovariectomy was also significantly faster than bilateral ovariectomy (*P* = 0.001). Conversely, in both adult lionesses and cubs, neither unilateral ovariectomy nor unilateral salpingectomy were significantly shorter than the respective bilateral procedure.

In comparing SPAS to MPAS, five cheetahs (SPAS group) were compared to five similarly sized lion cubs (MPAS group). Here the lion cubs, with a median body weight of 42 IQR 40–42 kg were heavier than the five cheetahs at 36 IQR 34–36 kg (*P* = 0.008). The abdominal wall however, was significantly thicker in cheetahs 11 mm (9.9–11.1 than in lion cubs 4.4 mm (3.8–4.4) (*P* = 0.008). All cubs were eight months old and were significantly younger than the cheetahs at 14 years (12–14) (*P* = 0.008). There was no difference in ovarian volume between the MPAS 316 mm^3^ (136–466) and SPAS groups 284 mm^3^ (163–293). Between one and six paraovarian cysts, measuring up to 18.9 mm on ultrasound, were associated with six of the ten ovaries in the five cheetahs, compared to none in lion cubs. Ovarian pedicle fat content in both species was zero and uterine tube and mesosalpinx morphology were similar laparoscopically. Median PPT for the SPAS group of 3 min (3–4) was significantly shorter compared to the MPAS group of 10 min (8–11) (*P* = 0.008) (Fig. 1) and median ST was also significantly shorter for SPAS at 8 min (7.5–8.5) than for MPAS ay 12 min (12–13) (*P* = 0.008). Resultant TST was significantly shorter for SPAS 20 min (17–22) compared to MPAS 35 min (30–36) (*P* = 0.008). There was however no difference in OPT between SPAS at 7 min (4–7) and MPAS group at 9 min (7–9) No ovaries were visible with patients in dorsal recumbency but all ovaries could be reached in the 45° oblique position in all patients.

There was no significant difference in mean volume CO_2_ required to complete ovariectomy (19.8 ± 8.1 L) or salpingectomy (20.7 ± 8.3 L) in lions using MPAS. The correlation between bodyweight and total volume of CO_2_ used in lions was significant (r_s_ = 0.867; *P* = 0.002) with a significant linear relationship between bodyweight and the total volume of CO_2_ required to complete sterilization (0.946). A regression formula, volume of CO_2_ = body weight in kg × 0.144 (coefficient) + 6.770 (constant), can be used to pre-determine the volume of CO_2_ that any specific lioness will require to acquire and maintain intra-abdominal pressures of 13 mmHg. For instance, a lioness that weighs 140 kg will require 140 × 0.144 + 6.770 = 26.93 liters of CO_2_ to complete surgery.

## Discussion

Salpingectomy was faster than ovariectomy in both adult lions and cheetahs since it is technically a simpler procedure. Total surgical time for sterilization in adult female cheetahs was faster than in lion cubs, which in turn was faster than in adult lions. OPT was the common denominator rendering TST shorter for salpingectomy in both groups. As expected, felids with larger body sizes took more surgical time to complete laparoscopic sterilization. Reported total surgery time in adult leopards [20] seem to be comparable to lion cubs in this study, with tigers [12] having the slowest of all known times documented for sterilization in large wild felids.

Median TST for salpingectomy in adult lions and cheetahs was significantly shorter than for ovariectomy. TST for cheetah laparoscopic ovariectomy using SILS in our study was 3.5 times faster compared to ovariectomy using SILS in two tigers, regarding the first case in this report as a learning experience (104 min) and using the second case as reference (84 min) and considering that specimen bags were used to retrieve ovaries in these tigers [12]. Similarly cheetah salpingectomy in our study was 1.3 times faster than the average time for salpingectomy performed in two leopards (33 and 25 min) also using SILS. Total surgical time for laparoscopic sterilization, irrespective of technique, in lion cubs was comparable to the two leopards and adult lionesses, which were faster than the two tigers but took longer than leopards and cheetahs. In dogs, laparoscopic ovariectomy has been reported to take 21 min for a single portal access and 19 min with two-portal access in one study [8]. In another dog ovariectomy study the application of one port, using a 5 mm vessel sealing device, took 30 min and two or three ports, using a 10 mm vessel sealing device, took 18 and 19 min respectively [9]. In domestic cats, laparoscopic ovariectomy using SILS takes 26 min [13]. In all of the abovementioned studies and our study, a bipolar vessel sealing device was used to seal and cut the ovarian pedicle.

PPT and ST were similar for the two procedures in adult lions, lion cubs and cheetahs, probably because the portal technique was standardized for both procedures within each respective group. Comparing unilateral salpingectomy to unilateral ovariectomy in each group, the extrapolated total time for unilateral salpingectomy was shorter than for unilateral ovariectomy for all animal groups in our study, but the difference was only statistically significant in cheetahs. Although bilateral salpingectomy was significantly faster than ovariectomy in adult lions, unilateral salpingectomy was not. This is probably because the significant advantage of shorter OPT during bilateral salpingectomy becomes diluted, within a prolonged TST, for the one-sided procedure in adult lions. Comparing the unilateral procedure to the respective bilateral procedure within each group, both unilateral ovariectomy and salpingectomy were significantly faster than the bilateral procedure in cheetahs however, this was not true in either adult lions or cubs. In the authors’ opinion, the role of unilateral salpingectomy in the population management of wild African felids holds promise and should be investigated further. Should unilateral salpingectomy be able to reduce litter size, this procedure could contribute to the management of population growth without disruption of social structures, especially in pride associated large carnivores such as lions.

In cubs, the use of two ports and an ovariectomy hook [6] or transabdominal suture [22–24] could be considered, since their thinner abdominal wall thickness may allow this application, in contrast to adult lionesses. However, this would probably not reduce surgery time, similar to dogs [9]. Clipping and surgical preparation of the surgical site for both MPAS and SPAS should be smaller and faster compared to the use of a transabdominal ovariectomy hook or suture. In all lion cubs the ovaries were small enough to retrieve via the 12 mm port of the MPAS, and application of this advantage should further reduce OPT for SPAS in patients with smaller ovaries. Although no cheetah cubs were incorporated in this study, surgery times in all cubs are anticipated to be similarly shorter, as was found in lions.

Utilization of a SPAS resulted in shorter total surgical time when compared to a MPAS. Interestingly, the operative time was not affected by utilization of standard straight instruments in SPAS when compared to MPAS. When considering the two different access systems, factors that could possibly affect PPT and ST are patient size and abdominal wall thickness. PPT and ST were significantly shorter for SPAS using a SILS port, despite SILS patients having a significantly thicker abdominal wall and a less conspicuous *linea alba*. In cheetahs, the *linea alba* was identifiable only in 50 % of patients [18] as opposed to lion cubs where it was consistently found [17]. These two factors seemed to be more influential in port placement and suturing than body weight, whereas age should not influence surgery times. OPT could possibly be influenced by ovarian size and paraovarian structures, ovarian pedicle fat content, uterine tube and mesosalpinx morphology and instrumentation. Ovarian size was comparable between populations. With MPAS used in lion cubs, all ovaries could be comfortably retrieved through the 12 mm cannula without enlarging the incision. However, the maximum ovarian size that can be retrieved through this cannula size needs to be determined. Since the SPAS incision is 2–3 cm in length [15, 18] even very large ovaries should be retrievable using this technique in wild felids. With MPAS, the 12 mm port has to be enlarged in order to retrieve larger ovaries, which requires subsequent temporary closure of the port in order to recreate a tight seal, for removal of the second ovary. All ovaries associated with paraovarian structures were comfortably retrieved with the SILS port in our study. Although ovarian size did not differ between the two groups, the presence of paraovarian cysts could complicate retrieval via a single 12 mm port of a MPAS similar to ovaries of larger size. The incidence of paraovarian cysts, as reported in cheetahs [25], may require the 12 mm port incision to be enlarged when using MPAS. Paraovarian cysts are much less common in lions [26], and have been reported in the tiger [12] however, not yet in leopards [20]. During some ovariectomy procedures, the tip of the uterine horn might also be inadvertently resected together with the ovary, resulting in bulkier tissue. Subsequent enlargement of a 12 mm port and recreating a tight seal in the three portal technique would therefore also prolong OPT and ST in these patients. In contrast, successful retrieval of smaller ovaries through the 5–12 mm cannula, without removing the SILS port, should further reduce OPT for SPAS. Furthermore, atraumatic retrieval of ovaries through the SILS port will preserve anatomic and histologic characteristics for further study and also permit oocyte harvesting from these ovaries [27]. Ovarian pedicle fat content as established by van Nimwegen for dogs [28] was negligible in both species in our study, did not differ as in dogs, and therefore did not influence surgery times as it might in canines [8, 15, 28], despite one study finding no influence on surgery time [15]. Uterine tube and mesosalpinx conformation was comparable between groups laparoscopically, which resulted in similar use of the vessel sealing device [17, 18] and therefore did not influence OPT or TST. The ease and comfort of using standard straight instruments [12, 15, 20] in our study were comparable for both access systems. This was supported by similar OPT for the two techniques. We did initially expect OPT to be longer for a single port compared to three ports because of reported instrument cluttering and loss of tri-angulation [11, 14]. However, this was not true either for removing ovaries or completing salpingectomy, despite the use of standard instruments. Possible reasons for shorter PPT and ST with SPAS compared to MPAS are that the SILS port required one incision only compared to three incisions and no Veress needle is required for SPAS. These should result in faster PPT compared to various MPAS’s that do make use of a Veress needle. Utilization of a Veress needle is associated with an increased risk of puncture of intra-abdominal organs and the Hasson technique has been used to prevent this complication [17]. Placement of a single port was subjectively found to be easier compared to using three separate ports. Because of non-parametric data, we could not adjust for species, bodyweight, abdominal wall thickness, and age or portal technique in a multiple regression model to determine the cause for shorter PPT, ST and TST using SPAS. Although the comparison between MPAS and SPAS in our study was made in a small cohort from two differing groups, our findings were consistent with those of a recent study by Gonzalez–Gasch and Monnet [29].

In both species patients had to be tilted 45º to the side in order to access the ovaries bilaterally. None of the patients required 90° true lateral position [8] or a Trendellenburg position [7, 29–31]. The spleen had to be manipulated in all patients to access the left ovary, similar to the leopard [20]. Despite using different theatre complexes for each respective project, similar surgical equipment and instrumentation were used, standardizing data collection in this respect. The initial ovaries were not randomized to minimize surgical variables.

The highlights in this paper are of importance when planning surgical expeditions to lion farms, conservation centers and especially national parks where predators roam free. In parks, where whole lion prides are typically darted at night using distress calls and bait, leaving a single lion of the pride undarted, holds severe risks for the team. The luxury of darting lionesses sequentially does not exist under these circumstances and all lions need to remain anaesthetized until every individual has been operated. The whole pride is then allowed to recover simultaneously. The number of females in the pride, coupled to TST (or TUPT), will therefore predict how long it will take before the last lioness is operated. Short, predictable surgery times are of the essence in the successful execution of surgical expeditions like this. Therefore, when permanent sterilization is considered as a management tool in population control of large wild felids, surgeries should be considered at a young age, salpingectomy will be faster in all adults and in cheetahs the unilateral procedure will be faster using SPAS.

The volume of CO_2_, to achieve and maintain intra-abdominal pressure at 13 mmHg throughout surgery, was higher in lions than required in cheetahs [18] and leopards [20] and can be accurately predicted using a regression formula. Typically, a 142 kg lioness will require 27 L of CO_2_ to complete either ovariectomy or salpingectomy using MPAS. This is extremely helpful when the supply of CO_2_, that needs to be transported in the field, can be calculated in advance. In a previous study, a poor correlation was found in cheetahs, probably because of very constant bodyweights in this cohort, but generally an adult cheetah requires 11.3 L to complete ovariectomy and 4.9 L for salpingectomy using SPAS [18]. Similarly, in cheetahs, a poor correlation was observed between bodyweight and TST. However, in lions, similar to CO_2_, TST was well correlated to bodyweight although the linear relationship was not strong. In cheetahs, removing the SILS port together with the first ovary required the entire peritoneal cavity to be re-insufflated, which significantly increased CO_2_ consumption. Using a trans-abdominal suture [6] or extraction bag [12] for the first ovary could have reduced CO_2_ requirements for the SPAS. However, the use of an extraction bag was likely to prolong surgery time [12] and using a suture would have obscured access to the fallopian tube for salpingectomy in this comparative study.

A limitation of this study was that the comparison of surgery times between groups are somewhat contrived, because two different portal techniques were used in lions and cheetahs. However, the authors found substance in comparing all known surgical aspects for laparoscopic sterilization of wild felids in one report.

## Conclusions

Surgery times for laparoscopic sterilization in wild felids are predictable and bilateral salpingectomy were faster than bilateral ovariectomy in both adult lionesses and cheetahs. In our study, both procedures were fastest in cheetahs and took the most time in adult lions, with cubs in between. For both ovariectomy and salpingectomy, the unilateral procedure was faster than the respective bilateral one in cheetahs using SPAS. Unilateral salpingectomy was also found to be faster than unilateral ovariectomy in cheetahs. Placement and closure of SPAS in cheetahs was faster and easier than a MPAS in lions and lion cubs, resulting in shorter TST for this access system in lions. Interference of standard straight instruments with SPAS was negligible and did not prolong surgery time.

## Abbreviations

MPAS: multiple portal access system
OPT: operative time
PPT: port placement time
SILS: single incision laparoscopic surgery
SPAS: single portal access system
ST: suturing time
TST: total surgical time
TUPT: total unilateral procedure time
